# A genetic screen of transcription factors in the *Drosophila melanogaster* abdomen identifies novel pigmentation genes

**DOI:** 10.1093/g3journal/jkae097

**Published:** 2024-05-31

**Authors:** Sarah J Petrosky, Thomas M Williams, Mark Rebeiz

**Affiliations:** Department of Biological Sciences, University of Pittsburgh, Pittsburgh, PA 15260, USA; Department of Biology, University of Dayton, Dayton, OH 45469, USA; Department of Biological Sciences, University of Pittsburgh, Pittsburgh, PA 15260, USA

**Keywords:** gene regulation, development, pigmentation, *Drosophila*, abdomen, CRISPR/Cas9

## Abstract

Gene regulatory networks specify the gene expression patterns needed for traits to develop. Differences in these networks can result in phenotypic differences between organisms. Although loss-of-function genetic screens can identify genes necessary for trait formation, gain-of-function screens can overcome genetic redundancy and identify loci whose expression is sufficient to alter trait formation. Here, we leveraged transgenic lines from the Transgenic RNAi Project at Harvard Medical School to perform both gain- and loss-of-function CRISPR/Cas9 screens for abdominal pigmentation phenotypes. We identified measurable effects on pigmentation patterns in the *Drosophila melanogaster* abdomen for 21 of 55 transcription factors in gain-of-function experiments and 7 of 16 tested by loss-of-function experiments. These included well-characterized pigmentation genes, such as *bab1* and *dsx*, and transcription factors that had no known role in pigmentation, such as *slp2*. Finally, this screen was partially conducted by undergraduate students in a Genetics Laboratory course during the spring semesters of 2021 and 2022. We found this screen to be a successful model for student engagement in research in an undergraduate laboratory course that can be readily adapted to evaluate the effect of hundreds of genes on many different *Drosophila* traits, with minimal resources.

## Introduction

The evolution of gene regulatory networks (GRNs) is thought to be a frequent mechanism for morphological diversity. These genetic programs underlie developmental processes for cells, tissues, and organs (Davidson 2006). In GRNs, transcription factors regulate their downstream target genes by binding to noncoding DNAs [cis-regulatory elements (CREs)] that control the transcriptional activity (enhancers) or repression (silencers) of those targets (Arnone and Davidson 1997; Levine and Davidson 2005). To identify changes within GRNs, a system is needed in which the essential transcription factors involved in a trait's development can be found and subsequently connected to CREs that control the expression of downstream genes.

The production of transgenic tools for genetic screens provides an avenue through which these essential transcription factors can be investigated. Genetic screens often utilize a loss-of-function (LOF) strategy. Modern techniques, such as RNA interference (RNAi) (Dietzl *et al*. 2007) and CRISPR/Cas9 (Bassett *et al*. 2013; Kondo and Ueda 2013; Yu *et al*. 2013; Port *et al*. 2014; Sebo *et al*. 2014), can quickly generate LOF via gene knockdown and gene knockout, respectively. Transgenic RNAi coupled with the Gal4/UAS system (Brand and Perrimon 1993; St Johnston 2002) allows for precise temporal and spatial control of gene knockdown and knockout and can bypass potential lethality of global knockdown or knockout (Perrimon *et al*. 2010; Heigwer *et al*. 2018; Meltzer *et al*. 2019). These LOF studies have been instrumental in finding components of GRNs, though these screens do not always capture the full impact of a gene's role in a phenotype. Some phenotypes are imperceptible when a gene is knocked down or knocked out (Rørth *et al*. 1998). In the *Drosophila melanogaster* genome, roughly 35% of genes with no known gene function have paralogs (Ewen-Campen *et al*. 2017), and thus, redundancy may render some phenotypes indiscernible. To overcome these complications and complement LOF studies, genes can be tested in gain-of-function (GOF) experiments. In GOF experiments, a gene of interest is ectopically expressed, resulting in over- or misexpression of that gene. GOF experiments can reveal additional nuance to a gene's function when combined with LOF results, and new relationships between genes and phenotypes can be identified that were not detected solely in LOF experiments. Finally, GOF experiments may reveal the potential paths that may exist to evolutionary change in other lineages, which may not be detected in LOF assays.

One model trait that has considerable potential to advance the understanding of GRNs in development and evolution is abdominal pigmentation in *D. melanogaster*. *Drosophila* species have evolved incredibly diverse pigmentation patterns that decorate the tergite plates covering the dorsal surface of the six large abdominal segments (Wittkopp *et al*. 2003), including phenotypes that are sexually dimorphic and which evolved from a monomorphic ancestor (Jeong *et al*. 2006; Hughes *et al*. 2020). Despite the remarkable diversity in abdominal pigmentation among *Drosophila* species, most transcription factors and pigmentation enzymes are highly conserved between *Drosophila* (Richards *et al*. 2005; Clark *et al*. 2007). Indeed, many cases of pigment evolution have been connected to mutations in gene regulatory sequences of the pigment network (Rebeiz and Williams 2017), although the binding transcription factors that mediate these mutational effects largely await discovery.

Previously, a LOF genetic screen with transgenic RNAi lines that targeted over 500 unique *D. melanogaster* transcription factors was performed (Rogers *et al*. 2014), which revealed 20 novel transcription factors whose reduced expression altered the pattern of abdominal pigmentation. For some of the factors, their effects were shown to influence the activity of multiple enhancers in this pigmentation GRN. Relatedly, another study employed a yeast 1-hybrid approach to identify 125 factors that had the ability to bind to the CRE for the pigmentation enzyme gene *yellow* (Kalay *et al*. 2016). Of these 125 transcription factor genes, RNAi knockdown of 32 resulted in altered tergite pigmentation to some detectable degree.

The Transgenic RNAi Project (TRiP) at Harvard Medical School previously generated transgenic RNAi lines for LOF experiments (Perkins *et al*. 2015). This project has recently developed a transgenic CRISPR/Cas9 approach that can be used to knockout or overexpress genes in a spatially and temporally controlled manner (Zirin *et al*. 2020). In this study, we present results from use of the TRiP CRISPR/Cas9 toolkit to knockout and overexpress candidate transcription factors in the abdominal midline, driven by the endogenous regulation of the *pannier* (*pnr*) gene (Calleja *et al*. 2000). Our screen included candidates identified in the prior RNAi screen (Rogers *et al*. 2014) and factors that may directly bind the *yellow* body CRE (Kalay *et al*. 2016). Gene knockouts in the transgenic CRISPR/Cas9 system largely recapitulated prior observations from RNAi knockdowns. By overexpressing these transcription factors in the abdominal midline, we demonstrated the utility of GOF experiments in elucidating gene functions and identified a candidate that, prior to this study, did not have a known role in tergite pigmentation patterning. We utilized these techniques in an undergraduate laboratory course, providing an authentic research experience to undergraduate students, and the positive outcomes demonstrate its utility as an educational tool.

## Methods

### Overexpression/knockout screen

**Fig. 1.**
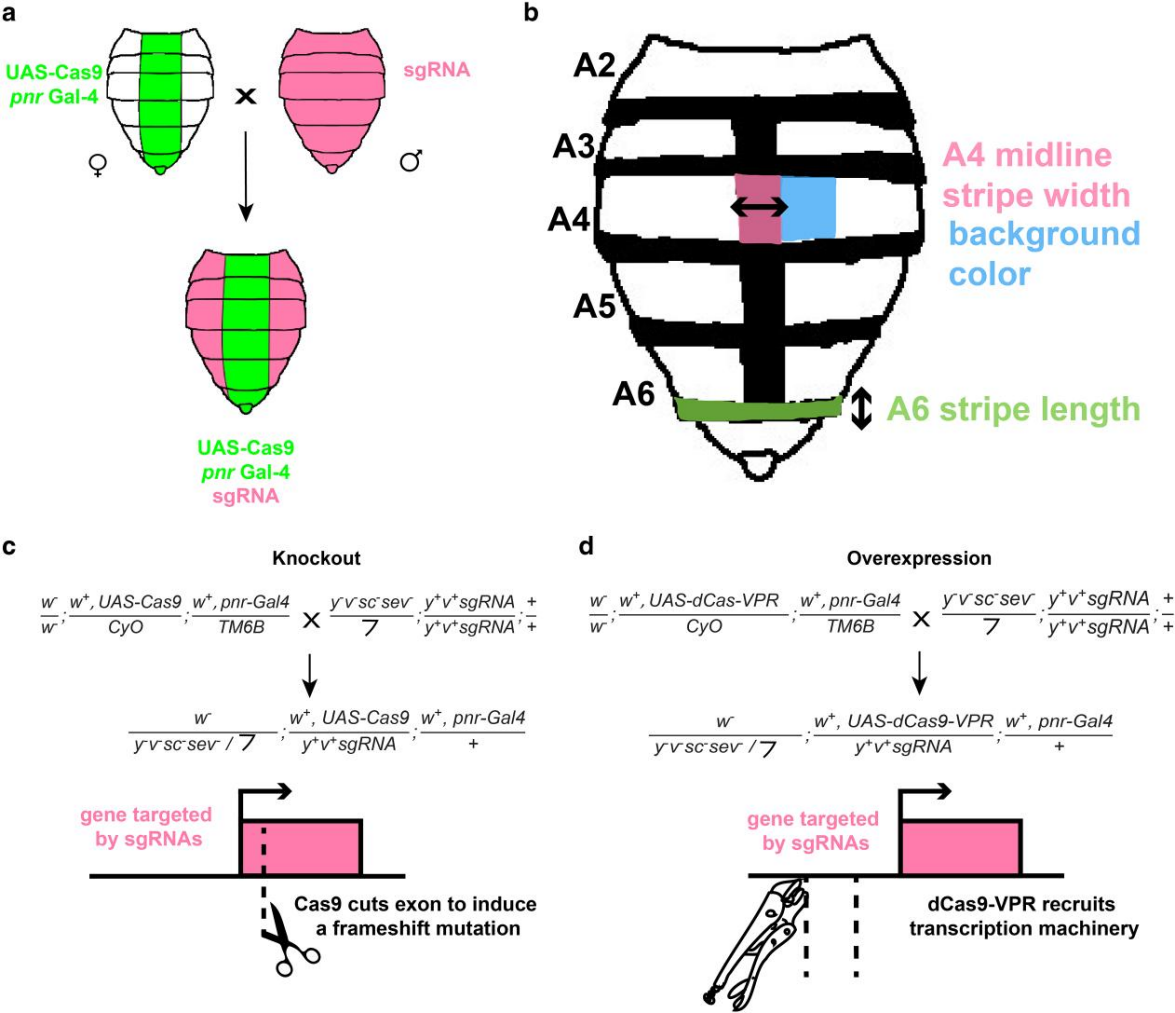
The TRiP transgenic gene editing system can be used for both overexpressing and knocking out genes of interest. a) Virgin females expressing either Cas9 or deactivated Cas9 fused to the VPR activation domain (dCas9–VPR) expressed in the abdominal midline driven by pnr were crossed to males with ubiquitous single guide RNAs. Progeny who received the Cas9 or dCas9–VPR–Gal4 driver and sgRNA were selected on the absence of dominant markers. b) Cartoon illustrates the 3 traits measured in this study: midline width, background color, and A6 stripe width. c) Genotypes of the parents and progeny in the knockout cross. In the knockout crosses, Cas9 can induce a frameshift mutation in the gene targeted by guide RNAs. These mutant gene alleles would produce a nonfunctional protein in the pnr expression domain. d) Genotypes of the parents and progeny in the overexpression cross. In the overexpression crosses, dCas9–VPR binds the promoter for a gene targeted by guide RNAs, recruiting transcription machinery to the gene of interest and ectopically expressing the gene in the pnr expression domain.

Fly lines were generated as a part of the Harvard Medical School TRiP (Zirin *et al*. 2020). All lines were acquired from the Bloomington Stock Center (see Supplementary Table 1 for stock numbers and lines). For the knockout crosses, 6–8 virgin females with *UAS–Cas9* and *pnr–Gal4* were crossed to 1–2 males with ubiquitously expressed guide RNA transgenes (Fig. 1c). In the conditional knockout progeny, Cas9 cleaves the target site as directed by the guide RNAs from the male parent that can induce a frameshift mutation upon repair in the protein coding sequence of the first or second exon (Fig. 1c). This results in a functional knockout of the targeted transcription factor in the midline of the abdomen, where *pnr* is expressed. For the overexpression crosses, 6–8 virgin females from a *pnr*–Gal4 driver line that additionally possesses a UAS-regulated deactivated Cas9 fused to the activator domain VP64–p65–Rta (dCas9–VPR) were crossed to 1–2 males possessing a pair of guide RNA transgenes (Fig. 1d). In the overexpression progeny, midline-expressed dCas9–VPR recruits transcriptional activation machinery to the promoter region near the transcription start site of the target gene as directed by the guide RNAs (Fig. 1d). This results in the ectopic expression of the targeted transcription factor in the midline. Both knockout and overexpression crosses used the same *pnr–Gal4* construct (Fig. 1a). All crosses were raised at 25°C.

### Imaging and analysis

The progeny from the crosses were transferred to new vials after eclosion. After culturing at 25°C for 7–9 days, flies were dissected by removing the wings and the legs, mounted on a slide covered with double-sided sticky tape, and imaged using a Leica M205C stereo microscope with a DFC425 camera. For each cross, around 10 male and 10 female abdomens per cross were mounted and imaged. Each abdomen was imaged under the same lighting conditions with an LED ring light. Extended focus brightfield images were generated using the Leica Montage package. The images taken all had a white glare as the result of the ring light used in the imaging process. To avoid the impact of the glare on our calculations, the pixels comprising the glare were not included in our analysis.

We conducted statistical analysis on 3 traits in female flies only: the A6 stripe (green), the midline stripe in the A4 segment (pink), and the background coloration in the A4 segment (blue) (Fig. 1b). For pigmentation intensity measurements, images were converted to grayscale and analyzed using FIJI. The segment of interest was outlined with the freehand tool, and a mean light value (*L*) in the range of 0–255 was recorded. The segment intensity was calculated in units of percent (%) darkness using the following equation (Pool and Aquadro 2007):


(255−L)/255×100%.

In addition, the FIJI straight-line tool was used to measure the anterior–posterior length of the female A6 stripe and the horizontal width of the A4 midline stripe (Fig. 1b). We did not quantify these 2 traits for the knockout crosses, as these effects have already been published (Rogers *et al*. 2014; Kalay *et al*. 2016). Raw measurements can be found in Supplementary File 1.

Two sets of quantitative data were compared using a 2-tailed Student's *t* test. Boxplots were generated in R and are presented as jittered plots, with the center lines representing the medians and the borders of the box representing the 25th and 75th percentiles. The *P*-values were adjusted by a Bonferroni correction to account for multiple testing. This increased the significance threshold from <0.05 to <0.001. The 2-tailed Student's *t* test results can be found in Supplementary Table 2. All image analysis was performed on blinded samples to eliminate bias.

### TRiP in an undergraduate laboratory course

We had the students in BIOSCI 0351 Genetics Lab, an upper-level university laboratory course, in spring 2021 and spring 2022 participate in these experiments at the University of Pittsburgh. Thirty-five students were enrolled in the spring 2021 course, and 34 were enrolled in the spring 2022 course. Students were divided into groups of 4 or 5, with each group having 1 transcription factor gene and 1 positive control gene [*bric-a-brac 1* (*bab1*) for overexpression crosses and *doublesex* (*dsx*) for knockout crosses]. The students established 2 test gene crosses and 2 control crosses, phenotyped progeny, and analyzed images using ImageJ as described above. The students were asked to organize and maintain a laboratory notebook for this experiment. At the end of the laboratory course, the students presented their findings to the rest of the class.

See Table 1 for the course timeline and materials needed for the course. Student learning objectives and methods of assessments are outlined in Table 2.

**Table 1.** Requirements and timeline for the Genetics Laboratory course.

Personnel and materials		Timeline	
Professors	1–2	Week 1	Introduction to fly husbandry
Teaching assistants	1	Week 2	Visualizing CRISPR targets
Students	34	Week 3	Journal club on CRISPR/Cas9
Fly food	4–8 vials per cross per group, plus vials to maintain stocks	Week 4	Primary literature search on gene
Fly stocks	1 sgRNA and 1 driver per group of 4	Week 5	Journal club on CRISPR/Cas9 in *Drosophila*
Brightfield microscope	Ideal: 1 per student Minimal: 1 per student group	Week 6	Setting up CRISPR cross
Microscope camera	1 per microscope	Week 7	Lab notebook check
Computers with FIJI	Ideal: 1 per student Minimal: 1 per student group	Week 8	Journal club on CRISPR in nonmodel organisms
		Week 9	Score progeny from CRISPR/Cas9 cross, TA mounts, and image flies
		Week 10	Ethics of CRISPR discussion
		Week 11	Analyzing image data, beginning poster presentation
		Week 12	Designing poster, wrapping up image analysis
		Week 13	Poster session, final lab notebook grading

**Table 2.** Learning objectives for the Genetics Laboratory course.

	Learning outcomes	Assessments
Knowledge	Articulate the molecular mechanisms of CRISPR/Cas9 actions	Journal discussions on CRISPR/Cas9 technology, weekly reflection paragraphs
	Frame student results in context of the current literature	Generate a discussion for poster presentation
	Examine ethical concerns regarding genome editing	Journal discussions on genome editing ethical concerns, weekly reflection paragraphs
Technical skills	Fly husbandry, including identifying virgin females, scoring based on sex and phenotype, and recognizing balancer chromosome phenotypes	Record their findings in a laboratory notebook
	Document lab activities reliably and consistently	Organize and maintain a laboratory notebook
Analytical skills	Develop hypotheses based on research into primary literature	
	Use ImageJ to measure properties of fly pigmentation, such as darkness and stripe width	Generate a results section for poster presentation
	Conduct statistical tests to determine significance of results	Generate a results section for poster presentation
Communication skills	Design graphics to convey experimental results	Final poster design
	Relay their experiments orally to their peers and colleagues	Final poster presentation

## Results and discussion

**Fig. 2.**
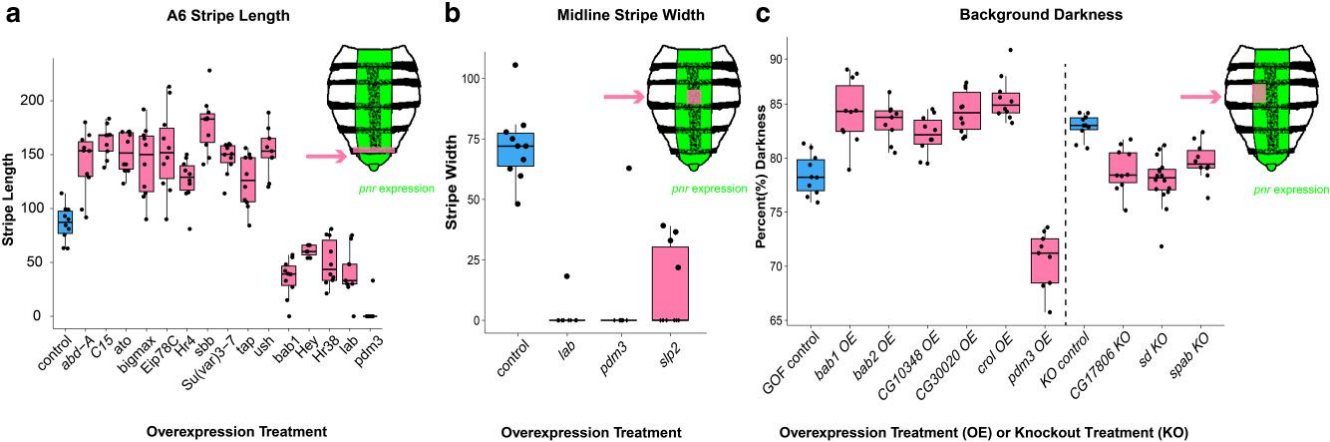
Changes among female flies to the anterior–posterior A6 stripe length, midline stripe width, and background pigmentation were observed in overexpression and knockout cross progeny. Two-tailed Student's *t* tests were used to compare targeted with control crosses, *P* < 0.001. a) Boxplot showing measurements of the A6 stripe in female flies compared with controls. Cartoon illustrates region of the fly measured (pink) and region affected by gene editing (green). b) Boxplot showing measurements of the midline stripe, assessed in the A4 segment of female flies, compared with controls. Cartoon illustrates region of the fly measured (pink) and region affected by gene editing (green). c) Boxplot showing calculated percent darkness of the A4 segment in female flies with a targeted transcription factor gene compared with controls. Cartoon illustrates region of the fly measured (pink) and region experiencing gene editing activity (green).

A total of 71 gene manipulations were performed, overexpressing 55 target and knocking out 16 transcription factor genes known to or suspected to function in the GRN for abdomen tergite pigmentation patterning and development. All transcription factor genes tested in this assay had previously been identified in RNAi screens (Rogers *et al*. 2014; Kalay *et al*. 2016). In Rogers *et al*. (2014), the transcription factor genes were chosen from the Drosophila Transcription Factor Database (Adryan and Teichmann 2006; Pfreundt *et al*. 2010), while Kalay *et al*. (2016) surveyed a collection of transcription factors fused to the Gal4 protein (Hens *et al*. 2011). Twenty-one of the overexpression crosses and 7 of the knockout crosses resulted in a phenotype that differed significantly from the control crosses. Some of the factors tested had detectible effects in more than one trait. For instance, *pdm3* resulted in the loss of the A6 and midline stripes and reduced pigmentation in background coloration (Fig. 2). Of the 8 genes for which we conducted both a GOF and LOF cross, none had detectible effects in both treatments. Representative images of progeny from the 9 knockout crosses and 34 overexpression crosses with no detectible phenotypic difference from the wild-type pigmentation patterns can be found in Supplementary Figs. 1 and 2, respectively.

The patterns in the *Drosophila* abdomen are largely determined by the presence or absence of 3 key enzymes, Yellow, Tan, and Ebony. Yellow is required to produce black melanin from dopamine that is present in the dark cuticle of the abdomen (Nash 1976; Wright 1987; Walter *et al*. 1991 ;Wittkopp *et al*. 2002; Drapeau 2003; Jeong *et al*. 2008; Hinaux *et al*. 2018). Tan and Ebony are both involved in catecholamine synthesis, with Ebony converting dopamine to beta-alanyl dopamine (Wittkopp *et al*. 2002, 2003; Richardt *et al*. 2003) and Tan reversing this reaction (True *et al*. 2005). These enzymes are expressed in patterns, with the dark producing enzymes Yellow (Wittkopp *et al*. 2003) and Tan (Jeong *et al*. 2008) localized in the stripes, midline, and male A5/A6 tergites, while Ebony is restricted to lighter cuticle patches (Rebeiz *et al*. 2009). The factors we identified may be involved in patterning the midline, either by repressing Tan and Yellow or promoting the dark pigment producing enzymes.

### Transcription factors that affect segment A5/A6 pigmentation

In some *Drosophila* species, the pigmentation in the A5 and A6 segments is sexually dimorphic. This trait is recently evolved (Gompel and Carroll 2003) and is thought to evolve from a monomorphic ancestor (Kopp *et al*. 2000; Jeong *et al*. 2006; Hughes *et al*. 2020). A number of transcription factors have been implicated in shaping the male-specific melanic A5–A6 pigmentation. The Hox genes *abdominal-A* (*abd-A*) and *Abdominal-B* (*Abd-B*) are expressed in the abdominal segments A2–A7 and A5–A7, respectively, and their expression is controlled by the *iab2-8* cis-regulatory elements (Akbari *et al*. 2006). *Abd-B* promotes the activity of the pigmentation enzymes *yellow* directly via binding sites in its cis-regulatory element and promotes *tan* indirectly (Jeong *et al*. 2006, 2008; Camino *et al*. 2015; Liu *et al*. 2019). The transcription factor genes *bab1* and *bric-a-brac 2* (*bab2*) play a large role in the sexual dimorphism of this trait by regulating *yellow*, a gene that encodes a pigmentation enzyme that produces black melanin (Kopp *et al*. 2000; Couderc *et al*. 2002; Salomone *et al*. 2013; Roeske *et al*. 2018). In turn, *bab1/2* expression is activated by *Abd-B*, and the sex-specific isoforms (DsxF and DsxM) of the transcription factor gene *doublesex* (*dsx*) regulate *bab1/2* in a sexually dimorphic pattern: DsxF activates *bab1/2* in females, and DsxM represses *bab1/2* in males (Williams *et al*. 2008). To capture additional genes that affect this sexually dimorphic pattern, we measured the length of the A6 stripe in the female progeny from our crosses.

We identified 18 factors whose altered expression results in a significant effect on pigmentation in the A5 and A6 abdominal segment tergites in either males or females. Of these 18 factors, we measured the length of the A6 stripe in female flies and detected a quantifiable difference between overexpression treatment and control flies (Fig. 2a and Table 3). It is important to note that pigmentation in the female A6 segment exhibits temperature-dependent plasticity (Gibert *et al*. 2000). To minimize the effect of environmental factors on the development of female pigmentation, all crosses were raised at 25°C. All 18 of these factors were significantly different from control flies post Bonferroni correction (Supplementary Table 2).

**Table 3.** Summary of the numerical values associated with A4 midline stripe width, A6 stripe length, and background darkness in overexpression treatments.

*Treatment (overexpression)*	A4 midline stripe (mean)	A6 stripe length (mean)	Percent darkness (mean)
*control*	72.11	86.30	79.24
*ab*	68.38	104.43	76.71
*abd-A*	175.4	142.75	81.32
*ato*	59.57	151.90	78.79
*bab1*	79.26	35.56	84.22
*bab2*	72.40	72.13	83.33
*bigmax*	65.44	150.06	80.15
*Br140*	69.05	104.96	79.00
*brm*	48.39	79.47	79.82
*C15*	80.71	162.40	80.87
*caup*	63.98	132.23	79.65
*CG10348*	60.79	58.84	82.16
*CG1233*	72.66	106.64	79.75
*CG9650*	84.42	174.30	79.52
*CG30020*	69.57	125.45	84.28
*CG33695*	76.37	118.65	79.80
*chinmo*	81.74	120.50	78.95
*crol*	90.08	115.81	85.45
*dsx*	53.41	63.05	81.79
*Eip78C*	92.23	153.85	82.18
*fru*	58.00	109.87	82.61
*Gsc*	99.83	125.23	79.99
*hb*	61.95	118.64	77.07
*Hey*	58.38	60.92	78.89
*Hr4*	69.67	126.49	81.30
*Hr38*	69.79	50.16	79.45
*Hr78*	61.59	100.20	76.81
*hth*	64.94	123.65	82.88
*ind*	73.88	113.75	76.65
*jing*	59.17	135.50	79.42
*lab*	1.80	39.74	79.40
*lmd*	74.48	120.06	79.28
*M1BP*	67.56	103.55	79.93
*Mad*	59.46	108.40	79.79
*MBD-like*	74.49	109.92	77.80
*Met*	69.94	113.00	79.74
*Mi2*	63.94	95.38	78.83
*nej*	65.27	97.99	80.12
*otp*	85.47	112.83	80.33
*pdm3*	7.01	0.00	70.63
*pita*	59.77	96.88	78.51
*pnt*	82.23	126.10	77.95
*sbb*	106.23	177.80	78.44
*scrt*	74.60	97.91	79.41
*slp2*	13.01	115.36	75.68
*Sox102F*	58.91	126.60	80.14
*Ssrp*	72.38	110.44	81.08
*Su(var)3-7*	78.05	146.90	80.05
*Su(z)12*	67.54	102.11	80.57
*tap*	69.70	125.28	80.66
*Tip60*	63.44	94.29	79.83
*tx*	74.81	117.51	80.38
*unpg*	0.00	160.00	84.96
*ush*	77.78	153.00	80.88

All numerical measurements were done in female flies. Background darkness has been converted to percent darkness as described in the *Methods* section. All measurements can be found in Supplementary File 1.

**Fig. 3.**
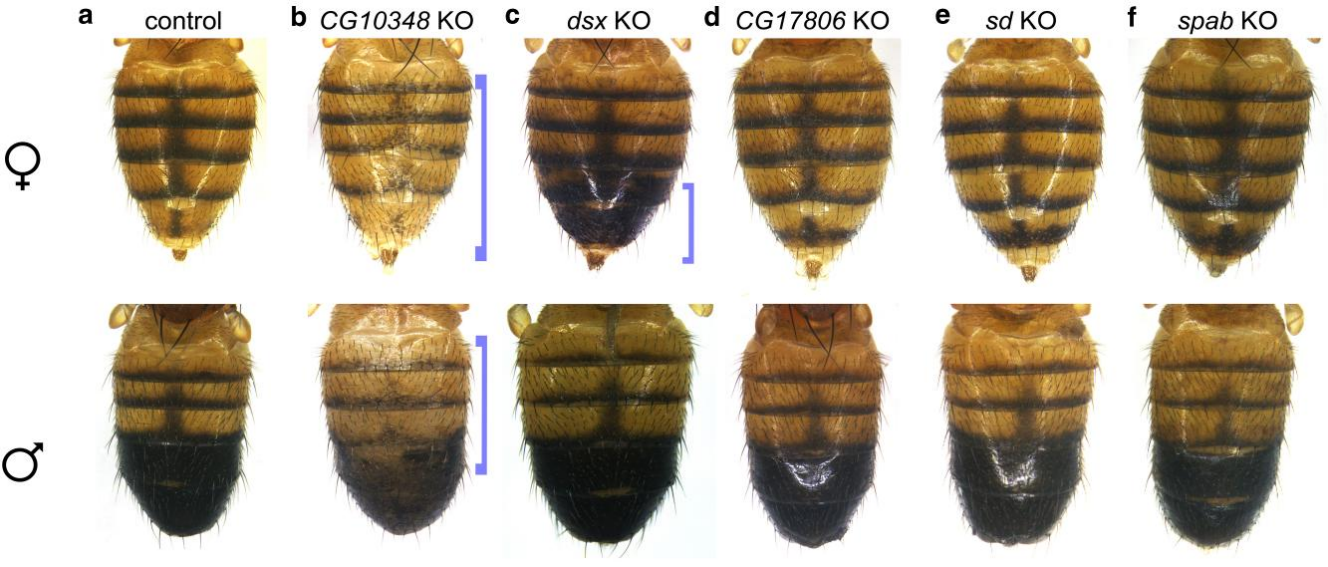
Noteworthy knockout tergite pigmentation phenotypes. Progeny of knockout crosses. Blue brackets highlight some notable phenotypes that were seen after imaging multiple samples, but are not representative of quantitative data. a) Knockout control abdomens. b–f) Gene knockouts featured here are b) *CG10348*, c) *dsx*, d) *CG17806*, e) *sd*, and f) *spab*. Knockouts *for CG10348* and *dsx* demonstrate decreased pigmentation in the midline and increased pigmentation in the female A5/A6 regions, respectively. *CG17806*, *sd*, and *spab* knockouts resulted in shifts in background coloration. All other knockout crosses did not have significant phenotypes in the areas measured. KO, knockout.

**Fig. 4.**
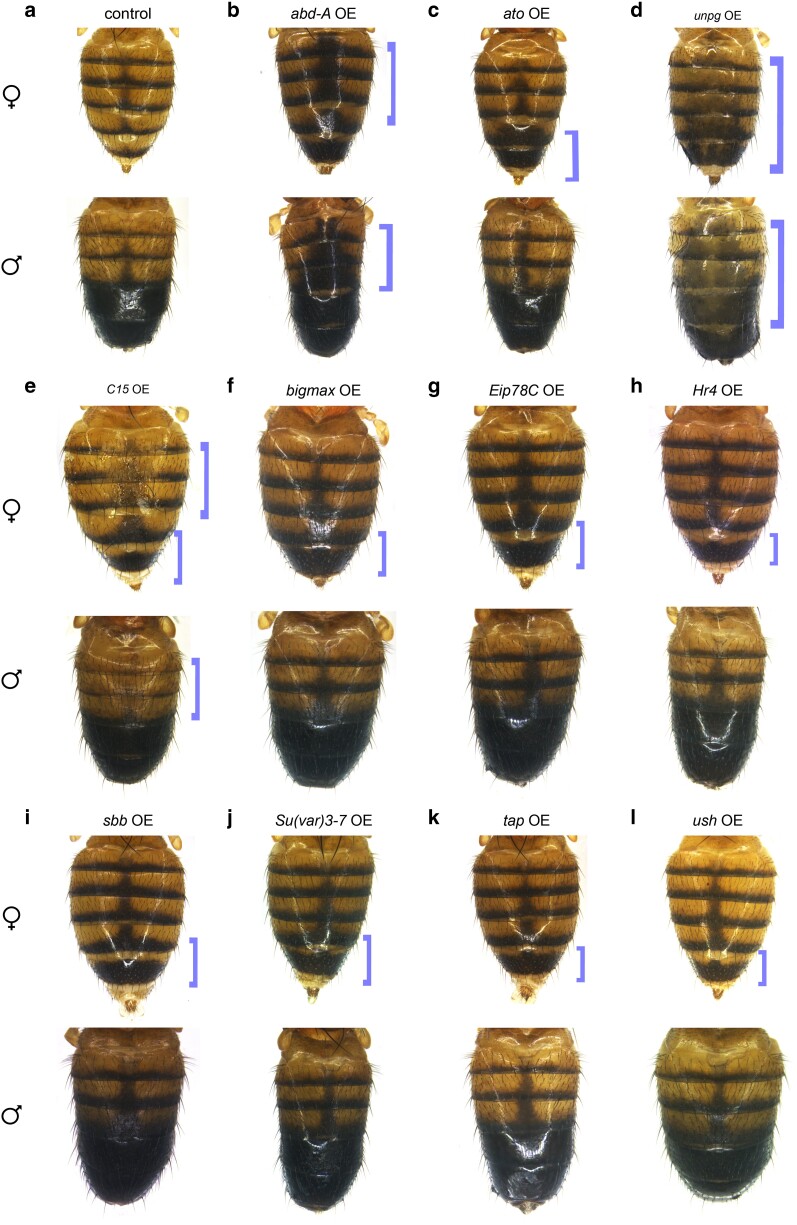
Overexpression phenotypes with an increase of melanic pigmentation. Progeny of overexpression crosses. Blue brackets highlight some notable increases in dark pigmentation that were observed after imaging multiple samples, but are not representative of quantitative data. a) Overexpression control abdomens. b–l) Overexpressed genes featured here are b) *abd-A*, c) *ato*, d) *unpg*, e) *C15*, f) *bigmax*, g) *Eip78C*, h) *Hr4*, i) *sbb*, j) *Su(var)3-7*, k) *tap*, and (l) *ush*. OE, overexpression.

Of these 18 transcription factor genes, 12 were identified as melanic pigment promoters, with LOF phenotypes from 1 cross including reduced melanic pigmentation and GOF phenotypes from 11 crosses including increased melanic pigmentation. Six of these transcription factor genes were previously identified in an RNAi screen (Rogers *et al*. 2014): *abd-A*, *CG10348*, *Hormone receptor 4* (*Hr4*), *scribbler* (*sbb*), *target of Poxn* (*tap*), and *unplugged* (*unpg*). *CG10348* (Fig. 3b), when knocked out, was consistent with the RNAi knockdown reported in Rogers *et al*. When overexpressed, *abd-A* (Fig. 4b), *Hr4* (Fig. 4h), *sbb* (Fig. 4i), and *tap* (Fig. 4k) all resulted in increased melanic pigmentation in the female A6 segment, while *unpg* overexpression resulted in melanic pigment that appeared more diffuse yet expanded in area (Fig. 4d). In Rogers *et al*., when knocked down, the transcription factor genes *abd-A*, *Hr4*, *sbb*, and *unpg* were found to reduce pigmentation in the A5 and A6 segments, and *tap* affected the thorax. The novel results are therefore consistent with the prior observations and thereby strengthen the inferred roles for these transcription factors acting as promoters of the melanic pigment patterning and development.

The other 6 transcription factor genes that were shown here to cause increased pigmentation in the female abdomen were previously identified in Kalay *et al*. (2016) as potential direct regulators of *yellow*: *atonal* (*ato*; Fig. 4c), *bigmax* (Fig. 4f), *C15* (Fig. 4e), *Ecdysone-induced protein 78C* (*Eip78C*; Fig. 4g), *Suppressor of variegation 3-7* (*Su(var)3-7,*Fig. 4j), and *u-shaped* (*ush*; Fig. 4l). When overexpressed, increased melanic pigmentation formed in the female A5 and A6 segments. This is consistent with the prior study (Kalay *et al*. 2016), as these factors resulted in reduced pigmentation when knocked down. The transcription factor genes *bigmax* (Fig. 4f) and *Suppressor of variegation 3-7* (*Su(var)3-7*; Fig. 4j), when overexpressed, increased pigmentation in the female A5 and A6 segments. In the prior study (Kalay *et al*. 2016), when knocked down, these factors had no effect on pigmentation, despite being identified as potential direct regulators of the pigmentation enzyme *yellow*. This suggests that, although knockdown of these factors has no effect on pigmentation in *D. melanogaster* lab strains, these factors may promote dark pigmentation when expressed in the abdomen, possibly by activating the expression of *yellow*.

**Fig. 5.**
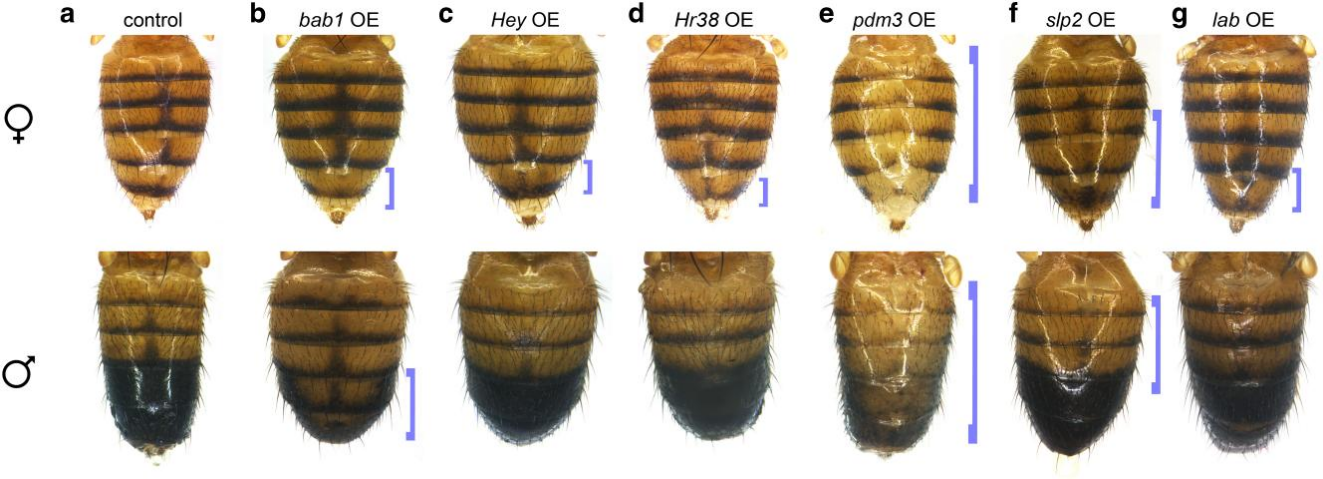
Overexpression phenotypes with a decrease in melanic pigmentation. Progeny of overexpression crosses. Blue brackets highlight some notable decreases in dark pigmentation that were observed across multiple samples, but are not representative of quantitative data. a) Overexpression control abdomens. b–g) Overexpressed genes featured here are b) *bab1*, c) *Hey*, d) *Hr38*, e) *pdm3*), f) *slp2*, and g) *lab*.

The remaining 6 transcription factor genes were implicated as repressors of the melanic pigmentation, including well-characterized transcription factor genes like *bab1* (Fig. 5b) and *dsx* (Fig. 3c). Additional factors with compelling phenotypes were *Hairy/E(spl)-related with YRPW motif* (*Hey*; Fig. 5c), *Hormone receptor-like in 38* (*Hr38*; Fig. 5d), *labial* (*lab*; Fig. 5g), and *pou domain motif 3* (*pdm3*; Fig. 5e), which, when overexpressed, resulted in reduced melanic pigmentation. The transcription factor genes *bab1*, *dsx*, and *pdm3* have verified roles in the patterning of the A5 and A6 segments. The transcription factors Bab1 and Bab2 repress *yellow* in a dimorphic pattern, due to the notable absence of *bab1/2* expression in the male A5 and A6 abdominal segment epidermis (Kopp *et al*. 2000; Couderc *et al*. 2002; Salomone *et al*. 2013; Roeske *et al*. 2018). This dimorphic pattern is controlled by Abd-B and Dsx, in which the DsxF isoform activates Bab in females and the DsxM isoform represses Bab in males (Williams *et al*. 2008). The factor *pdm3* has been implicated as a potential indirect repressor of *yellow* (Yassin *et al*. 2016; Liu *et al*. 2019). Our results are consistent with prior studies that investigated these three genes as repressors of the endogenous melanic pigment formation.

### Transcription factors that affect midline patterning

In *D. melanogaster*, both male and female flies exhibit a darkly pigmented vertical stripe in the dorsal–ventral midline of the abdomen. This pattern is at least partially controlled by Decapentaplegic (Dpp) signaling. Ectopic Dpp activity promotes increased pigmentation in the dorsal–ventral midline of the abdomen (Kopp *et al*. 1999, 1997). To assess the effects of additional factors on the width of the midline stripe, we measured the width of the stripe in the A4 segment.

We identified 6 transcription factor genes that impacted the width of the midline stripe in the A4 segment (Fig. 2b and Table 3). When overexpressed, the transcription factor genes *lab* (Fig. 5g), *pdm3* (Fig. 5e), and *sloppy paired 2* (*slp2*; Fig. 5f) produced a thinner or nonexistent midline stripe. Two of the tested transcription factor genes, *C15* (Fig. 4e) and *unpg* (Fig. 4d), when overexpressed, resulted in faded pigmentation in the midline region, but the boundaries of the midline appear to be wider than wild type. Notably, *C15* also promotes dark pigment in the female A5 and A6 tergites, indicating that it acts as both a promoter and repressor of melanic pigmentation. Although *unpg* is involved in both A5/A6 pigmentation and midline pigmentation, the pigment in flies overexpressing *unpg* in the dorsal midline appears diffuse compared with the wild-type pattern. Another factor, *CG10348*, resulted in a reduced midline stripe when knocked out.

The *slp2* result is notable because *slp2* previously had no known role in pigmentation. It had been identified in a yeast 1-hybrid screen as capable of binding to the *yellow* wing + body *cis*-regulatory element, but *slp2* LOF experiments did not produce detectible effects on abdominal pigmentation (Kalay *et al*. 2016). In this GOF assay, we observed that *slp2* could reduce pigmentation in the midline when overexpressed (Fig. 5f). These results indicate that *slp2* either has a redundant function in abdominal pigmentation, which would make detecting its effects difficult in LOF screens, or that *slp2* is not endogenously expressed in the *pnr* domain of the abdominal cuticle in *D. melanogaster* but can nevertheless repress it. Much of our knowledge on the pigmentation network comes from experiments with *D. melanogaster*, so the identification of new factors like *slp2* may lead to insights in the pigmentation networks of other *Drosophila* species.

### Transcription factors that affect background coloration

In addition to the sexual dimorphism in the A5 and A6 segment tergites and the patterning of the midline stripes, we were interested in evaluating the changes to the lighter (yellow–brown) colored cuticle, or background coloration, of the progeny. Background pigmentation has been implicated in adaptation of *D. melanogaster* populations. In African *D. melanogaster* populations, background pigmentation is correlated with altitude, with populations at higher altitudes exhibiting darker background pigmentation (Pool and Aquadro 2007; Bastide *et al*. 2014). Previously, the gene *ebony* was found to underlie the increased dark background pigment in a Ugandan population (Rebeiz *et al*. 2009), and single-nucleotide polymorphisms in regulatory regions for *tan* and *bab1* have been associated with pigmentation variation in European populations (Bastide *et al*. 2013). To capture factors that may affect background coloration, we measured the difference in background coloration intensity in our crosses.

We identified 9 transcription factor genes that had subtle effects on the background coloration (Fig. 2c and Table 3). In many cases, these shifts in coloration are subtle, shifting the background coloration as little as 3–5%. When knocked out, the factors *CG17806* (Fig. 3d), *scalloped* (*sd*; Fig. 3e), and *space blanket* (*spab*; Fig. 3f) shifted the background pigmentation slightly lighter, indicating these genes may have normally function as promoters of darker background coloration. When overexpressed, the transcription factor genes *bab1/2*, *CG10348*, *CG30020*, and *crol* shifted the background pigmentation slightly darker, while *pdm3* shifted the background pigmentation lighter. Some of these alterations are counterintuitive. For example, *bab1/2* is characterized as a pigment repressor, while overexpression of *bab1/2* in this cross resulted in darker background pigmentation, rather than lighter. These results might suggest a more complex role for Bab1 and Bab2 in the operation of the pigmentation GRN. However, this counterintuitive outcome might be due to variation in the genetic backgrounds of the guide RNA lines, as the shifts in background pigmentation are subtle, with less than 5% difference in pigment intensity compared with the control.

These screens are useful for generating candidate genes underlying adaptive phenotypes. In other African populations, notably one from Fiche, Ethiopia, genome sequencing data have implicated multiple genomic regions as contributing to differing phenotypes in background coloration (Bastide *et al*. 2016). Indeed, many of the genes tested, including *bab1/2*, *CG10348*, *dsx*, *Ecdysone-induced protein 74EF* (*Eip74EF*), *pdm3*, *Suppressor of variegation 2-10* [*Su(var)2-10*], and *unpg* among others, fall under QTL peaks associated with pigmentation variation described by Bastide *et al*. (2016). This screen and future screens may reveal causative genes underlying these adaptive phenotypes. In addition, GOF screens can illuminate additional paths that adaptation can take, as the candidates identified in GOF screens that were not identified in LOF screens of 1 species may have been important in the evolutionary diversification of related species.

### Transcription factors that alter development in the abdomen and thorax

**Fig. 6.**
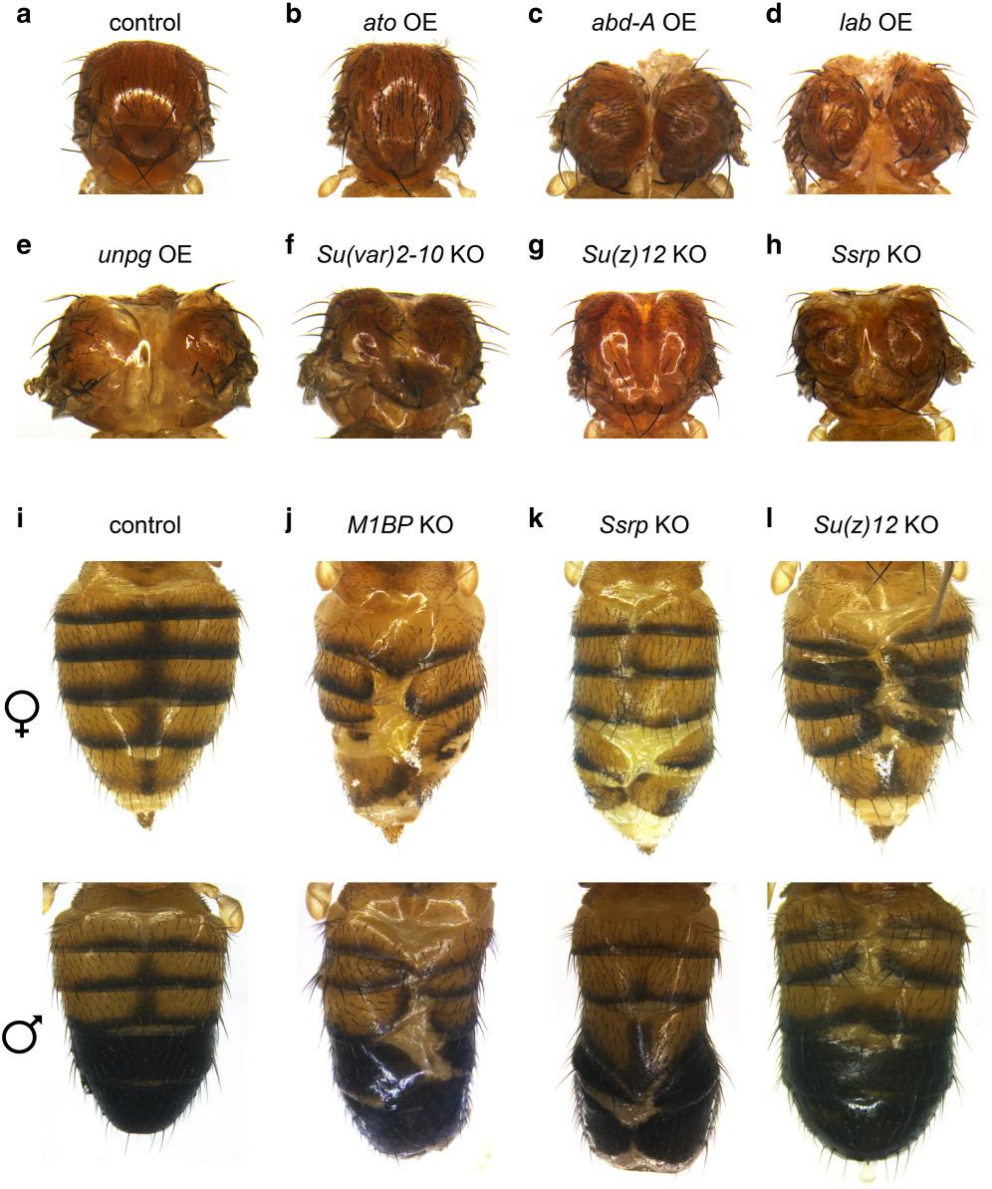
Defects in the development of the thorax and abdomen. a) Control thorax. b) The gene *ato* produces additional bristles on the thorax when overexpressed. c–e) When overexpressed, the genes c) *abd-A*, d) *lab*, and e) *unpg* produce a defect in the thorax. f–h) When knocked out, the genes f) *Su(var)2-10*, g) *Su(z)12*, and h) *Ssrp* produce a defect in the thorax. i) Control abdomens. j–l) When knocked out, the genes j) *M1BP*, k) *Ssrp*, and l) *Su(z)12* produce a defect in the midline of the abdomen.

Several factors affected the morphology of the thorax and the abdomen. The transcription factor genes *abd-A* (Fig. 6b), *lab* (Fig. 6d), and *unpg* (Fig. 6e), when overexpressed, produce flies with indented thoraxes. Two of these transcription factor genes, *abd-A* and *lab*, are homeotic genes that are responsible for proper segmentation and development of the abdomen and anterior thorax, respectively. *abd-A*, along with *Abd-B*, is part of the bithorax complex and is regulated by *trithorax* in proper development of the abdominal segments (Breen and Harte 1993). *lab* is part of the Antennapedia complex, which is responsible for the development of the head and anterior thoracic segments (Diederich *et al*. 1989 ).

The factor *ato*, when overexpressed, produces flies with additional bristles on the thorax (Fig. 6c), though it did not produce additional bristles in the abdomen. This may be due to differences in the developmental patterning of the thorax compared with the abdomen The factor *Su(var)2-10*, when knocked out, results in a slight indentation in the thorax (Fig. 6f). The factor *Motif 1 Binding Protein* (*M1BP*; Fig. 6j), when knocked out, produces flies with improperly developed tergites. The factors *Structure specific recognition protein* (*Ssrp*) and *Su(z)12* impact both the thorax and the abdomen when knocked out: the thoraces develop indentations (Fig. 6g and h), while the abdomens exhibit defects in tergite development (Fig. 6k and l). In addition to the developmental defects, *abd-A*, *ato*, *lab*, and *unpg* have effects on pigmentation when overexpressed, and *Su(var)2-10* affects pigmentation when knocked out.

### Efficacy of CRISPR/Cas9 in genetic screens

Prior LOF studies relied on RNAi technology, and we expected the results of our CRISPR/Cas9-mediated knockouts to be consistent with the outcomes of prior RNAi screens (Rogers *et al*. 2014; Kalay *et al*. 2016). The progeny from the knockout crosses in this study are largely congruent with the results from prior RNAi studies; however, some genes showed no detectible phenotypic difference from wild-type abdominal pigmentation, despite a measurable phenotypic effect in RNAi studies. Examples of this deviation include *Eip74EF*, Hr4, and *tango* (*tgo*) (Rogers *et al*. 2014).

These discrepancies may be due to the design of the transgenic lines. Transgenic CRISPR/Cas9 mediates gene knockout quite effectively: in the transgenic CRISPR/Cas9 library generated by Port *et al*. (2020), less than 10% of the generated transgenic lines produce insufficient target mutations, a marked improvement over current *Drosophila* RNAi libraries (Perkins *et al*. 2015). However, there are also some caveats in experimental design. For example, some transgenic knockout lines will encode 1 guide RNA sequence, while others encode 2 guide RNAs. Those encoding 2 guide RNA sequences may produce more conspicuous phenotypes compared with a line with only 1 guide RNA sequence (Xie *et al*. 2015; Yin *et al*. 2015; Port and Bullock 2016). We imaged 10 males and 10 females for as many crosses as possible to capture subtle phenotypes; however, it is possible that some transcription factor genes may nevertheless have subtle phenotypes below the threshold of detection in this assay. In some cases, such as the *dsx* knockout, the effectiveness of the knockout varied from individual to individual (Supplementary Fig. 3). Finally, it is worth noting that the Kalay *et al*. study (2016) used flattened cuticle preparations to measure phenotypes, which are likely more sensitive to subtle effects.

### Educational value of transgene-based genetic screens

In addition to the scientific value of the TRiP CRISPR/Cas9 system, this technique has much promise as an educational tool. Course-based undergraduate research experiences allow undergraduate students to engage in authentic research projects in a laboratory course setting (Auchincloss *et al*. 2014). These courses provide an accessible research experience to many students and promote engagement with hypothesis-driven research at all stages of the scientific process. CRISPR/Cas9 has been used for laboratory courses in *Drosophila* (Adame *et al*. 2016), bacteria (Pieczynski *et al*. 2019), yeast (Sehgal *et al*. 2018), frogs (Martin *et al*. 2020), and butterflies (Martin *et al*. 2020). Students have responded positively to research-based laboratory courses, compared with traditional laboratory courses (Martin *et al*. 2020). Incorporating CRISPR/Cas9 into laboratory courses provides scientific and educational value (Wolyniak *et al*. 2019), and projects designed using the TRiP toolkit can allow students to engage with this technology in most laboratory settings and pursue a wide variety of research questions with relative ease.

This screen was conducted as part of the Genetics Lab course, comprised of primarily sophomore and junior undergraduate students. In groups of 4 to 5, each student group was assigned an experimental transcription factor to either overexpress or knockout, as well as a positive control cross. For groups conducting a knockout assay, the positive control was *dsx*, while the positive control for the overexpression groups was *bab1*. These 2 controls had been tested prior to the start of the class to ensure that they would be effective positive controls. In spring 2021, the course had 7 student groups of 5. Five of those groups conducted overexpression assays for *CG10348*, *crol*, *Hr4*, *lmd*, and *unpg*, while the other 2 groups conducted knockout assays for *CG10348* and *Hr4*. In spring 2022, the course had 7 student groups of 4 and 1 group of 5. Six of those groups conducted overexpression assays for *ato*, *bab2*, *CG10348*, *Hr4*, *osa*, and *slp2*, while the other 2 groups conducted knockout assays for *CG10348* and *Hr4*.

In this approach, students are highly involved in the discovery process. The students began by searching for articles on their transcription factor and learned techniques for finding good sources and reading research articles effectively with the guidance of the instructors. The students were able to contribute to most portions of the experiment, even those who attended remotely or asynchronously for some meetings, and all students received data that they could analyze using FIJI.

We found that the results of this genetic screen were more productive than prior attempts to incorporate CRISPR/Cas9 into an educational experience with more laborious approaches involving germline editing. Although we focused on A6 pigmentation, midline patterning, and background coloration in this manuscript, the students were encouraged to measure additional traits and were not directed by the instructors to measure particular traits. More than half of the student groups identified significant changes from the control in at least 1 trait, and those that did not nevertheless produced useful negative data. We attribute the relative success of the educational TRiP screen to the ease with which these resources allow students to generate phenotypes and explore gene functions.

Similar projects can be implemented in undergraduate labs to provide an authentic research experience to undergraduate students. The materials needed for the project workflow are minimal, requiring only the fly stocks, fly food, and a way to anesthetize the flies and image body parts. This strategy can be applied to many structures using hundreds of genes.

In addition, this project has been implemented in both virtual and in-person formats. We designed these experiments to provide activities that students could participate in when class could not be fully conducted in person during 2021. Our setup allowed for 6 students to be in the room safely with the instructor and the teaching assistant. Two students from each of the 7 groups were able to attend lab in person for each class period. The virtual students focused on literature searches, while the in-person students set up the crosses. Both sets of students could fully participate in image and statistical analysis. When the class was fully in person in 2022, all students had the opportunity to participate in both the in-lab and virtual components. In both semesters, the mounting and imaging were carried out by the teaching assistant. Although this screen works better for the students when they are all in person, we found that it was simpler to adapt to a hybrid format than previous iterations of the class.

### Conclusion

The purpose of this study was to confirm previous knockdown experiments and survey the effects of pigmentation transcription factors when overexpressed in the abdominal midline. We used a transgenic CRISPR/Cas9 system to overexpress 55 transcription factor genes identified in prior RNAi screens as potential regulators of pigmentation enzymes. We identified 18 factors that affected A6 tergite pigmentation, 6 that affected midline stripe patterning, 9 that affected background pigmentation, and 8 factors that affected thorax and abdominal morphology (Table 4). While a number of these factors, including *abd-A*, *bab1/2*, and *dsx*, have been well characterized in prior studies, we were able to observe phenotypes in the abdomen caused by transcription factors that are not as well characterized in this developmental context, such as *C15*, *CG10348*, and *unpg*. We determined a role for new factors that previously had not been implicated in tergite pigmentation, such as *slp2*, and provided new candidates for pigmentation studies. GOF experiments, such as those conducted in this screen, can elucidate potential paths to evolutionary change, as the phenotypes observed in GOF experiments but not LOF experiments in 1 species may be important in other species. In addition, we used this technique to provide an authentic research experience to undergraduate students in a Genetics Laboratory course and found that this project workflow could be easily adapted for other university courses.

**Table 4.** Summary of observed phenotypes.

*Treatment*	A4 midline width		A6 stripe length		Background pigment	Defects	
	Males	Females	Males	Females		Thorax	Abdomen
*abd-A OE*	None	None	None	+	None	✓	None
*ato OE*	None	None	None	+	None	✓	None
*bab1 OE*	None	None	−	−	+	None	None
*bab2 OE*	None	None	None	None	+	None	None
*bigmax OE*	None	None	None	+	None	None	None
*C15 OE*	−	−	None	+	None	None	None
*CG10348 OE*	None	None	None	None	+	None	None
*CG10348 KO*	−	−	−	−	None	None	None
*CG30020 OE*	None	None	None	None	+	None	None
*crol OE*	None	None	None	None	+	None	None
*dsx KO*	None	None	None	+	None	None	None
*Hey OE*	None	None	None	−	None	None	None
*Hr38 OE*	None	None	None	−	None	None	None
*Hr4 OE*	None	None	None	+	None	None	None
*lab OE*	−	−	None	−	None	✓	None
*M1BP KO*	None	None	None	None	None	None	✓
*pdm3 OE*	−	−	None	−	−	None	None
*sbb OE*	None	None	None	+	None	None	None
*slp2 OE*	−	−	None	None	None	None	None
*Ssrp KO*	None	None	None	None	None	✓	✓
*Su(var)2-10 KO*	None	None	None	None	None	✓	None
*Su(var)3-7 OE*	None	None	None	+	None	None	None
*Su(z)12 KO*	None	None	None	None	None	✓	✓
*unpg OE*	+	+	−	+	None	✓	None
*ush OE*	None	None	None	+	None	None	None

Increases in pigmentation are represented by “+.” Decreases in pigmentation are represented by “−.”

## Data Availability

All data analyses and representative images are contained in this manuscript. All raw image files not featured in this manuscript and Supplemental material are available via FigShare (DOI 10.6084/m9.figshare.24123111): https://figshare.com/articles/dataset/Raw_Data_from_Manuscript_A_genetic_screen_of_transcription_factors_in_the_i_Drosophila_melanogaster_i_abdomen_performed_in_an_undergraduate_laboratory_course_/24123111.
